# Ilimaquinone Induces Apoptosis and Autophagy in Human Oral Squamous Cell Carcinoma Cells

**DOI:** 10.3390/biomedicines8090296

**Published:** 2020-08-20

**Authors:** Cheng-Wen Lin, Li-Yuan Bai, Jui-Hsin Su, Chang-Fang Chiu, Wei-Yu Lin, Wei-Ting Huang, Ming-Cheng Shih, Yu-Ting Huang, Jing-Lan Hu, Jing-Ru Weng

**Affiliations:** 1 Department of Medical Laboratory Science and Biotechnology, China Medical University, Taichung 40402, Taiwan; cwlin@mail.cmu.edu.tw; 2 Department of Biotechnology, Asia University, Wufeng, Taichung 41354, Taiwan; 3 Division of Hematology and Oncology, Department of Internal Medicine, China Medical University Hospital, Taichung 40447, Taiwan; lybai6@gmail.com; 4 College of Medicine, China Medical University, Taichung 40402, Taiwan; d5686@mail.cmuh.org.tw; 5 National Museum of Marine Biology and Aquarium, Pingtung 94450, Taiwan; x2219@nmmba.gov.tw; 6 Cancer Center, China Medical University Hospital, Taichung 40415, Taiwan; 7 Department of Pharmacy, Kinmen Hospital, Kinmen 89142, Taiwan; u8557006@gmail.com; 8 Department of Biological Science & Technology, I-Shou University, Kaohsiung 82445, Taiwan; c960099@gmail.com (W.-T.H.); mchshih@isu.edu.tw (M.-C.S.); 9 Department of Nutrition, I-Shou University, Kaohsiung 82445, Taiwan; ythuang@isu.edu.tw; 10 Department of Marine Biotechnology and Resources, National Sun Yat-sen University, Kaohsiung 80424, Taiwan; annavsbelle@yahoo.com.tw; 11 Doctoral Degree Program in Marine Biotechnology, National Sun Yat-sen University, Kaohsiung 80424, Taiwan; 12 Graduate Institute of Pharmacognosy, College of Pharmacy, Taipei Medical University, Taipei 11042, Taiwan

**Keywords:** ilimaquinone, OSCC, autophagy, apoptosis, p53

## Abstract

In this study, the anti-tumor activity of ilimaquinone (IQ), a sesquiterpene quinone isolated from marine sponge *Halichondria* sp., in oral squamous cell carcinoma (OSCC) cells, was investigated. IQ suppressed the viability of the OSCC cell lines SCC4 and SCC2095 with IC_50_ values of 7.5 and 8.5 μM, respectively. Flow cytometric analysis demonstrated that IQ induced caspase-dependent apoptosis in SCC4 cells and modulated the expression of several cell growth-related gene products, including Akt, p38, Mcl-1, and p53. Notably, p53 knockdown caused higher resistance to IQ’s anti-tumor activity. In addition, IQ increased reactive oxygen species generation, which was partially reversed by the addition of antioxidants. Furthermore, it triggered autophagy, as evidenced by acidic organelle formation and LC3B-II and Atg5 expression in SCC4 cells. Pretreatment with the autophagy inhibitor 3-methyladenine or chloroquine partially decreased IQ-induced apoptosis, suggesting that IQ induced protective autophagy. In summary, IQ has potential to be used in OSCC therapy.

## 1. Introduction

The incidence of oral squamous cell carcinoma (OSCC), which has a high mortality rate, has witnessed increases in developing counties, such as India [1,2]. In Asia, betel quid chewing, smoking, and alcohol consumption are the main OSCC risk factors [3]. Several mutated genes, including cyclin D1, p53, and Rb, and the dysregulation of molecular pathways have been associated with its progression [4]. In addition to surgery, current therapies include chemotherapy, radiotherapy, and immunotherapy [5]. However, the 5-year survival rate has remained at 50%, for the past decades [3], indicating the urgent need for new therapies.

For over 34 years, natural products have provided ~40% of developed FDA-approved therapeutic agents [6]. The source of some of these therapeutic agents is the oceans, which contain a rich biodiversity of marine organisms [7]. For example, trabectedin, an alkaloid isolated from a tunicate, has been used to treat advanced soft tissue sarcoma for over 10 years [8]. Additionally, since 2010, eribulin mesylate, originally developed from halichondrin B, a macrocyclic polyether obtained from marine sponges, was approved as metastatic breast cancer therapy [8,9]. Therefore, the ocean is a potential source of anti-tumor agents.

Among marine organisms, marine sponges produce several secondary metabolites, including quinones, steroids, alkaloids, terpenoids, and fatty acids, some of which possess drug-like characteristics [10]. Ilimaquinone (IQ), a sesquiterpene quinone isolated from marine sponges, exhibits a variety of biological activities, including anti-inflammatory [11], antitubercular [12], anti-HIV [13], and anti-tumor [14]. Previous studies have revealed that IQ interferes with protein transport by damaging the Golgi apparatus in cervical cancer cells [15]. Additionally, multiple studies have demonstrated that IQ induces apoptosis in several cancer cell lines, including prostate cancer, colon cancer, and multiple myeloma [16,17,18]. For example, it induces apoptosis through caspase-3 activation, and upregulates the expression of p53 and p21 in colon cancer cells [18]. By activating ROS-ERK/p38 MAPK-CHOP signaling, it also potentiates tumor necrosis factor-related apoptosis-inducing ligand (TRAIL)-induced apoptosis in colon cancer cells [14]. Furthermore, some studies have reported that IQ inhibits cancer cell growth by regulating β-catenin [17], GADD-153 [16], and p53 [18]. However, its anti-tumor activity in OSCC is still unclear. This study therefore investigated its anti-tumor effects in OSCC cells, as well as the underlying mechanism.

## 2. Materials and Methods

### 2.1. Reagents, Chemicals, Antibodies

The sponge *Halichondria* sp. was collected by scuba diving at a depth of 10 m from coral reefs off the Coast of Pingtung County, in 2016. Voucher specimen has been deposited in the National Museum of Marine Biology and Aquarium, Taiwan (specimen No. SP2016-01). The frozen sponge *Halichondria* sp. material (450 g, wet weight) was freeze-dried, and the resulting material (80 g) was then minced and extracted exhaustively with ethyl acetate (EtOAc) (1 L) six consecutive times with the duration of one week each time. The EtOAc crude extract was evaporated under reduced pressure to afford a residue (3.66 g). Then, the residue was separated over a silica gel by column chromatography to obtain IQ (811 mg). The identity and purity of IQ were verified by a comparison of its 1D and 2D proton nuclear magnetic resonance (NMR) spectral data (Figures S1–S5) with available literature [19]. All agents were dissolved in dimethyl sulfoxide (DMSO), diluted in culture medium, and added to cells at a final DMSO concentration of 0.1%. Other chemicals and reagents were obtained from Sigma-Aldrich unless otherwise noted.

### 2.2. Cell Culture

SCC4 human oral squamous cell carcinoma cells were purchased from Japanese Collection of Research Bioresources (Tokyo, Japan), and SCC2095 human oral squamous cell carcinoma cells were kindly provided by Professor Susan R. Mallery (The Ohio State University). All cell lines were cultured in dulbecco’s modified eagle medium (DMEM)/F12 medium (Gibco, Grand Island, NY, USA) which containing 5 mg/mL of penicillin, 10% fetal bovine serum (FBS) (Gibco), and 5 mg/mL streptomycin at 37 °C in a humidified incubator containing 5% CO_2_.

### 2.3. Cell Viability Analysis

Cell viability of IQ was assessed by using the 3-(4,5-dimethylthiazol-2-yl)-2,5-diphenyltetrazolium bromide (MTT) assays [20]. Briefly, cells (5 × 10^3^) were seeded and incubated in 96-well, flat-bottomed plates in 10% FBS-supplemented medium for 24 h and were exposed to compound at indicated concentrations for different time intervals. After removing the medium, it was replaced by 200 μL of 0.5 mg/mL MTT in 5% FBS-medium, and cells were incubated at 37 °C for 4 h. The medium was removed and the reduced MTT dye was solubilized in 200 μL/well DMSO. Absorbance was determined with a SPECTROstar Nano spectrophotometer (BMG LABTECH, Ortenberg, Germany) at 570 nm.

### 2.4. Flow Cytometric Analysis

Cells (2 × 10^5^/3 mL/per well) were plated in a six-well plate and treated with DMSO or the indicated concentration of IQ for 48 h [20]. For apoptosis evaluation, after being washed twice with phosphate-buffered saline (PBS), followed by spinning at 1200 rpm for 5 min, and cells in 500 μL PBS were stained with 3 μL annexin V-fluorescein isothiocyanate (FITC) and 5 μL propidium iodide (PI) at room temperature for 15 min according to the vender’s protocols (BD Pharmingen, San Diego, USA) and analyzed by using an Attune NxT flow cytometer (ThermoFisher Scientific, Waltham, MA, USA). For reactive oxygen species (ROS) production determination, cells were washed with PBS twice and stained with the fluorescence probe 5-(and-6)-carboxy-2′,7’-dichlorodihydrofluoresceindiacetate (carboxy-DCFDA, 5 μL, Invitrogen) at 37 °C for 30 min and analyzed by a flow cytometer.

### 2.5. Immunoblotting

Protein was collected from the cells after various treatments. For Western blots, a previously described procedure was applied [20]. The immunoblotting was performed with primary antibodies recognizing p^473^Ser-Akt (#9271), Akt (#9272), PARP (#9542), cleaved caspase-9 (#7237), p^180^Thr/^182^Tyr-p38 MAPK (#9215), p38 MAPK (#9212), HIF-1α (#3716), Mcl-1 (#39224), survivin (#2802), LC3B(#2775), p^15^Ser-p53 (#9284), and p53 (#2527) all from Cell Signaling Technologies (Beverly, MA, USA); procaspase-8 (MAB4708) from Millipore; Bax (ab7977) from Abcam (Cambridge, UK); Bcl-2 (Sc-509) from Santa Cruz Biotechnology (CA, USA); Atg5 (GTX62601) from GeneTex (Irvine, CA, USA); β-actin (A5316) from Sigma-Aldrich (St. Louis, MO, USA). Immunoblotted bands were visualized by an enhanced chemiluminescence reagent (GE Healthcare Bioscience, NJ, USA) and system (FUSION SoLo S, Deutschland, Germany) using secondary antibodies (Santa Cruz Biotechnology, Santa Cruz, CA, USA).

### 2.6. Detection of Autophagosome Formation with Acridine Orange

To detect the presence of acidic vesicular organelles (AVOs), IQ-treated cells were stained with acridine orange (1 μg/mL) for 15 min and washed with PBS. Then, the cells were examined under a fluorescence microscope [21].

### 2.7. Transient Transfection

SCC4 cells (2 × 10^5^/3 mL) were seeded in each well of a six-well plate. After a 24 h incubation, cells were transfected with p53 shRNA (TRCN0000003755, the National RNAi Core Facility, Taipei, Taiwan) using Fugene HD reagent (Roche) for another 24 h. Then, the cells were treated with IQ for 48 h and collected the protein for Western blotting.

### 2.8. Statistical Analysis

The data are presented as the mean of at least three replicates. Statistical significance was performed by Student’s *t* test, and *p*-values (* *p* < 0.05, ** *p* < 0.01) were considered as significant.

## 3. Results

### 3.1. IQ Decreases the Viability of OSCC

**Figure 1:**
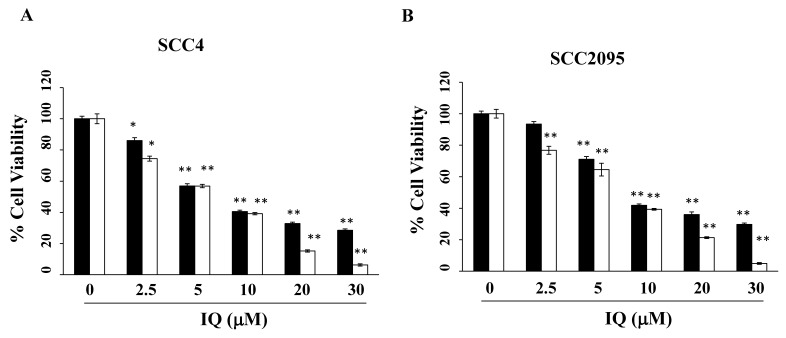
Effect of ilimaquinone (IQ) on the viability of oral cancer cells (SCC4 and SCC2095). (**A**) SCC4 and (**B**) SCC2095. The cells were seeded onto 96-well plates, incubated for 24 (■) and 48 h (□), treated with IQ for 48 h and then cell viability was detected using the 3-(4,5-dimethylthiazol-2-yl)-2,5-diphenyltetrazolium bromide (MTT) assays. Points, means; bars, S.D. (*n* = 3–6). * *p* < 0.05 and ** *p* < 0.01 vs. the control group.

Using MTT assays, the anti-viability effect of IQ was assessed in two OSCC cell lines: SCC4 and SCC2095. As shown in Figure 1A,B, IQ decreased cell viability in a concentration- and time-dependent manner. IQ concentrations of 7.5 and 8.5 μM decreased cell viability by 50% after 48 h in SCC4 and SCC2095 cells, respectively. Given that, after treatment, the IC_50_ value of IQ in SCC4 cells was lower than that in SCC2095, the former was used in subsequent experiments.

### 3.2. IQ Induces Caspase-Dependent Apoptosis in SCC4 Cells

**Figure 2:**
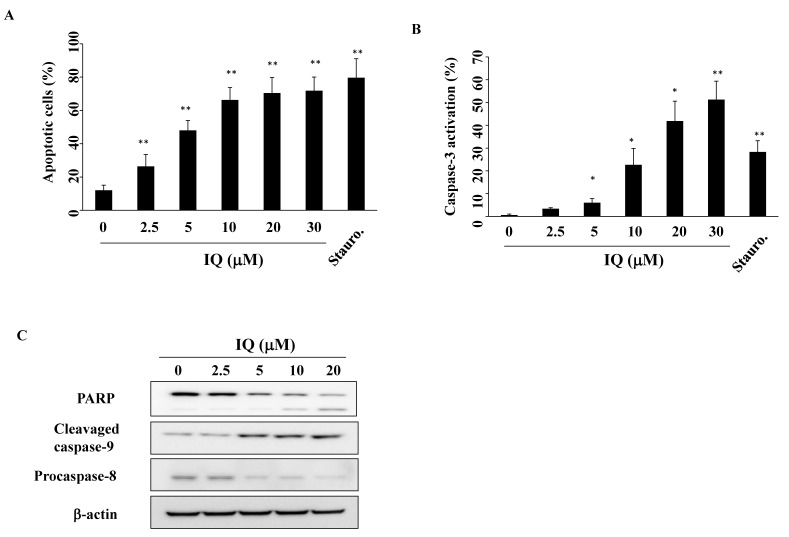
Effect of ilimaquinone (IQ) treatment on apoptosis. (**A**) The percentage of apoptotic cells (Q2 + Q4) after dimethyl sulfoxide (DMSO) vehicle, IQ, or 50 nM staurosporine (Stauro.) treatment for 48 h. SCC4 cells were treated with either DMSO, IQ, or Stauro. in 5% fetal bovine serum (FBS)-supplemented DMEM/F12 medium for 48 h and stained with propidium iodide (PI)/annexin V. Columns, mean; bars, S.D. (*n* = 4). ** *p* < 0.01 vs. the control group. (**B**) Concentration-dependent effect of IQ on caspase-3 activation. Staurosporine (Stauro.; 25 nM) was used as the positive control. Columns, mean; bars, S.D. (*n* = 3). * *p* < 0.05 and ** *p* < 0.01 vs. the control group. (**C**) Expression of PARP, cleavage caspase-9, and procaspase-8 in SCC4 cells after 48 h treatment in 5% FBS-supplemented DMEM/F12 medium.

To determine whether apoptosis resulted from IQ-mediated cell death, SCC4 cells were treated with IQ for 48 h, and assessed using propidium iodide (PI)/Annexin V analysis. As shown in Figure 2A, IQ increased the percentage of apoptotic cells in a concentration-dependent manner (staurosporine as a positive control). To confirm the involvement of caspase activation, SCC4 cells were treated with IQ for 48 h, and analyzed for activated caspase-3 using flow cytometry (Figure 2B). This treatment increased activated caspase-3 levels, from 0.6% in the control group to 6.0%, 22.7%, 41.8%, and 51.3% in SCC4 cells treated with 5, 10, 20, and 30 μM of IQ, respectively (staurosporine as a positive control). Compatible with flow cytometric staining findings, IQ treatment led to the activation of caspase-8, caspase-9, and poly (ADP-ribose)polymerase (PARP) cleavage in a concentration-dependent manner (Figure 2C). These results confirm that IQ induces caspase-dependent apoptosis in SCC4 cells.

### 3.3. IQ Modulates Akt and p53 Expression in SCC4 Cells

**Figure 3:**
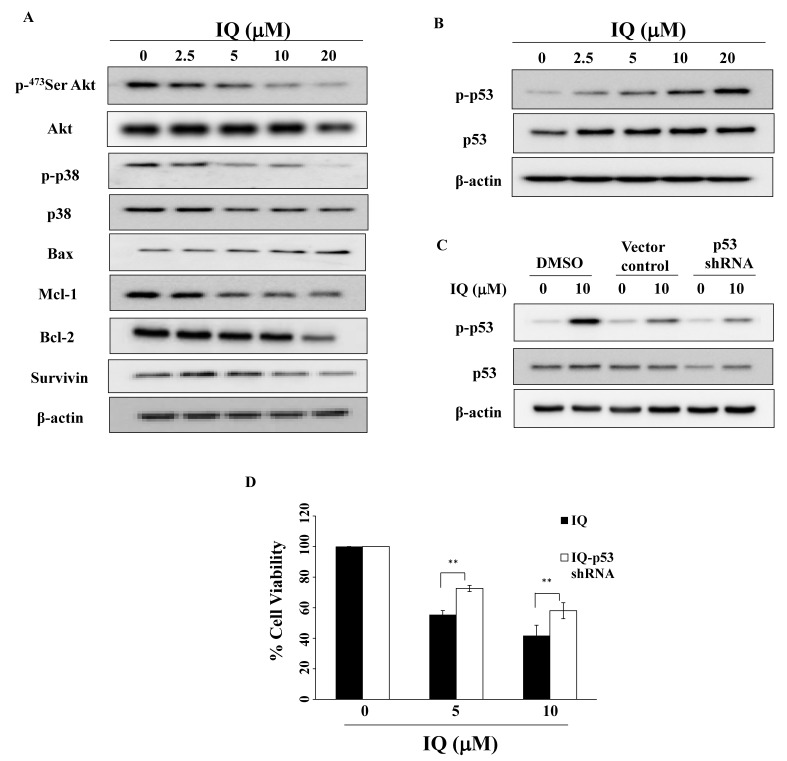
Effect of IQ on various biomarkers in SCC4 cells. (**A**) Phosphorylation/expression of Akt, p38, Bax, Mcl-1, Bcl-2, and survivin in SCC4 cells. Cells were treated with IQ for 48 h, and cell lysates were immunoblotted as described in Materials and Methods. (**B**) Expression of p-p53 and p53 of IQ in SCC4 cells. (**C**) Western blotting of p-p53 and p53 in SCC4 cells transiently transfected with a control vector or p53 shRNA for 24 h. (**D**) IQ-induced cell viability in p53 knockdown SCC4 cells. After incubation, cells were analyzed using the MTT assays. Columns, mean; bars, S.D. ** *p* < 0.01.

Previous studies demonstrated that Akt and p38 activation mediates OSCC migration and that blocking their signaling increased the sensitivity of OSCC cells to chemotherapeutic agents [22,23,24,25]. This study reveals that, in OSCC cells (Figure 3A), IQ downregulated the expression of p-Akt, p-p38, and the metastasis prognostic marker HIF-1α [26]. Consistent with caspase-dependent apoptosis, IQ increased proapoptotic protein Bax expression in a concentration-dependent manner, accompanied by a parallel decrease of the anti-apoptotic proteins Mcl-1 and Bcl-2, and the apoptotic protein inhibitor survivin [27] (Figure 3A).

Previous studies have revealed that Bax and apoptotic protease activating factor (APAF-1) could through the transcriptional activity of p53, a tumor suppressor gene, be implicated in OSCC [28,29]. Western blotting demonstrated that IQ increased p53 phosphorylation in SCC4 cells (Figure 3B). To further investigate the role of p53 in IQ-mediated cytotoxicity, cells were transiently transfected with p53 shRNA or a control vector, and then treated with IQ for 48 h. As shown in Figure 3C,D, p53 knockdown caused higher resistance to IQ’s viability inhibition activity.

### 3.4. IQ Increases Reactive Oxygen Species (ROS) Generation in SCC4 Cells

**Figure 4:**
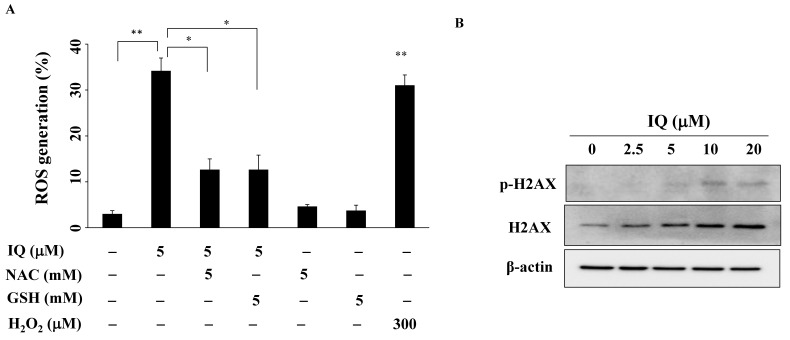
IQ increases reactive oxygen species (ROS) production. (**A**) Statistical analysis of ROS generation in SCC4 cells after the treatment with only 5 μM of IQ for 3 h or in combination with 5 mM of *N*-acetylcysteine (NAC) or 5 mM of glutathione (GSH). Cells were stained with carboxy-DCF-DA and analyzed using flow cytometry. Data are expressed as mean ± S.D. (*n* = 3). * *p* < 0.05 and ** *p* < 0.01. (**B**) IQ-induced H2AX phosphorylation/expression in SCC4 cells.

Growing evidence shows that ROS generation in oxidative stress causes DNA damage, which contributes to areca nut-induced OSCC carcinogenesis, and chemotherapy resistance [30,31]. After treatment with IQ, ROS levels in SCC4 cells were examined. Figure 4A shows that ROS generation was significantly higher in the IQ-treated groups, compared with the control group (H_2_O_2_ was the positive control), and could be partially reversed by pretreatment with the antioxidant *N*-acetylcysteine (NAC) or glutathione (GSH) for 15 min. Furthermore, in SCC4 cells, IQ upregulated the expression and phosphorylation of H2AX, a DNA damage response marker (Figure 4B).

### 3.5. IQ induces Autophagy in SCC4 Cells

**Figure 5:**
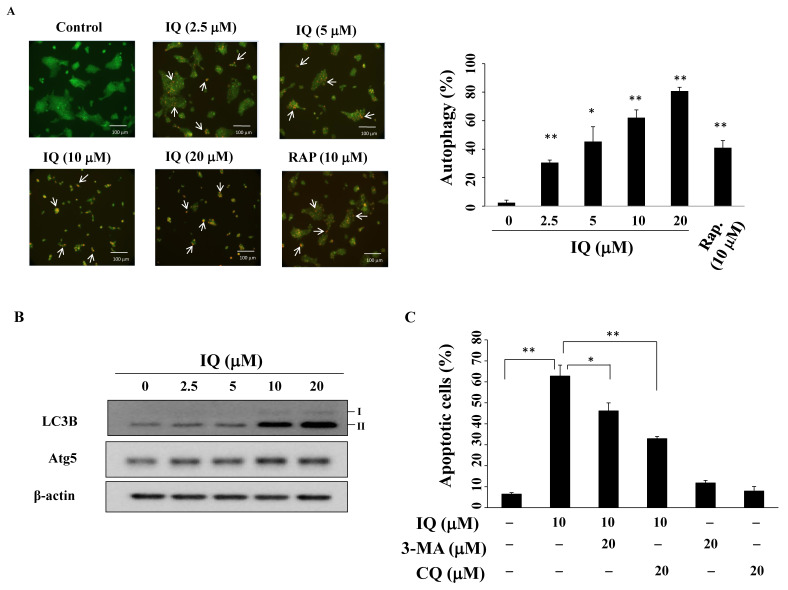
Ilimaquinone (IQ) induces autophagy. (**A**) Left panel, SCC4 cells were treated with DMSO vehicle, IQ, or rapamycin (RAP) for 24 h, harvested and stained with acridine orange, and examined under a fluorescence microscope; arrows, acidic vesicular organelles (AVOs). Right panel, statistical analysis of AVOs in IQ-treated SCC4 cells. Columns, mean; bars, S.D. (*n* = 3). * *p* < 0.05 and ** *p* < 0.01 vs. the control group. (**B**) LC3B-II and Atg5 expression in SCC4 cells after IQ treatment for 48 h. (**C**) SCC4 cells were pretreated 20 μM of 3-methyladenine (3-MA) or 20 μM of chloroquine (CQ) for 15 min, followed by incubation with 10 μM IQ for 48 h and dual staining with propidium iodide (PI)/annexin V- fluorescein isothiocyanate (FITC). Percentages in the graphs are representative of cell percentage in the respective quadrants (*n* = 3). Columns, means; bars, S.D. * *p* < 0.05 and ** *p* < 0.01.

Autophagy, which can promote cell survival or death, plays a dual role in cancer therapy [32]. To investigate autophagy, acidic vesicular organelles (AVOs) were observed under a fluorescence microscope. During autophagy, the volume of the cellular acidic compartment is an autophagy marker that can be visualized using acridine orange staining [33]. After the treatment with IQ, an increase in the number of cytoplasmic AVOs in SCC4 cells was observed (Figure 5A, left panel; rapamycin as the positive control) and the ratio of acridine orange-positive cells increased in a concentration-dependent manner (Figure 5A, right panel). Western blotting demonstrated that IQ increased the expression of the autophagic biomarkers LC3B-II [34] and Atg5 [35] (Figure 5B). The autophagic inhibitor 3-methyladenine (3-MA) or chloroquine (CQ) was used to determine the role of autophagy in IQ-induced cell death in SCC4 cells. As shown in Figure 5C, pretreatment with either 3-MA or CQ decreased the percentage of IQ-induced apoptotic cells, indicating that IQ induced protective autophagy instead of autophagic cell death.

## 4. Discussion

Natural marine products have served as a source of potential pharmaceutical agents for centuries [36]. This study demonstrates that IQ, a marine sponge metabolite, can inhibit OSCC growth by mediating Akt downregulation, modulating Bcl-2 family and p53, increasing ROS, and inducing autophagy.

Multiple studies have revealed that some anti-cancer agents exert their effect by activating apoptosis, which is the major pharmacological mechanism in cancer therapy, including OSCC [36,37,38]. During apoptosis, the mitochondria and the activation of death receptors lead to the activation of caspase cascades [38,39]. Previous studies have reported that IQ induces apoptosis via death receptor-mediated extrinsic pathway activation and the downregulation of anti-apoptotic proteins such as Bcl-2 and Bcl-xL, which are related to mitochondria-mediated apoptosis in colon cancer cells [14]. Furthermore, multiple genes are involved in the control of apoptosis. For example, the Bcl-2 family’s oncogenic potential has been demonstrated in oral tumorigenesis [40]. This study reveals that IQ induces PARP cleavage and caspase-dependent apoptosis in SCC4 cells. In addition to the modulation of Mcl-1 and Bcl-2 expression, IQ treatment increased the level of Bax and decreases that of survivin.

The MAPK/p38 and PI3K/Akt signaling are involved in the regulation of the growth, metastasis, and apoptosis of OSCC, and the inhibition of Akt and p38 blocked the migration of OSCC cells and [23,41]. Akt activation has been correlated with the poor prognostic factor with local recurrence and five-year survival rate for OSCC [22]. Neoh et al. reported that flaccidoxide-13-acetate from the soft coral reduces the cell migration of bladder cancer cells through the down-related Akt/mTOR pathway [42]. Our results demonstrate that IQ downregulated the phosphorylation of Akt and p38 in OSCC cells. It has been reported that IQ increased p38 activation to sensitize TRAIL-induced apoptosis in colon cancer cells [14]. It is possible that there are different additional mechanisms regulated by IQ or the difference in cell lines used. Furthermore, our study shows that IQ inhibited the expression of HIF-1α, a key molecule in the regulation of hypoxia, which contributes to the tumor metastasis of OSCC [43].

One of the genes activated in response to several stress stimuli such as oncogene activation and DNA damage is p53 [44]. Immunohistochemical analysis revealed that there are over 60% OSCC patients with higher expression of p53, and the concentration of p53 of OSCC patients in saliva was significantly higher than in healthy participants [45,46]. Previous studies have shown that IQ inhibits cell growth in colon cancer by increasing p-p53 [18]. The results of this study indicate that treatment with IQ did not only increase p53 phosphorylation, but also partially protected against IQ-induced cytotoxicity after p53 knockdown in SCC4 cells.

Increased ROS levels act as an intracellular signal transducer, promoting autophagy, which is the pharmacological mechanism of some anticancer drugs currently in clinical use [47,48]. Do et al. reported that IQ increases ROS generation in TRAIL-induced apoptosis in colon cancer cells [14]. Similarly, we found that the antioxidant NAC or GSH partially rescued IQ-induced ROS production in SCC4 cells.

In tumor cells, autophagy can be activated by cellular stress stimulation, induced by starvation, a hypoxic environment, or cytotoxic agents [49]. In colon cancer and glioblastoma cells, IQ reportedly induced autophagy through p53 activation [18,50]. Similarly, our results show that IQ induces autophagy, as evidenced by the accumulation of autophagosomes in the cytoplasm, and LC3B-II and Atg5 expression in SCC4 cells. Interestingly, in the presence of the autophagic inhibitor 3-MA or CQ, the percentage of IQ-induced apoptotic cells decreased, suggesting that IQ induces protective autophagy in SCC4 cells.

## 5. Conclusions

In conclusion, IQ exerts anti-tumor effects either by inducing apoptosis and autophagy or by increasing ROS, revealing its translational potential as a new therapeutic strategy in OSCC treatment.

## Supplementary Materials

The following are available online at https://www.mdpi.com/2227-9059/8/9/296/s1, Figure S1: ^1^H NMR spectrum of ilimaquinone in CDCl_3_ at 400 MHz; Figure S2: ^13^C NMR spectrum of ilimaquinone in CDCl_3_ at 100 MHz; Figure S3: HMQC spectrum of ilimaquinone in CDCl_3_; Figure S4: HMBC spectrum of ilimaquinone in CDCl_3_; Figure S5: ^1^H–^1^H COSY spectrum of ilimaquinone in CDCl_3_.
