# Supplementary material for: Ilimaquinone Induces Apoptosis and Autophagy in Human Oral Squamous Cell Carcinoma Cells
Source: Biomedicines. 2020 Aug 20;8(9):296. doi: 10.3390/biomedicines8090296 (PMC7555415; doi:10.3390/biomedicines8090296)
Supplement: Supplementary file 1 [file biomedicines-08-00296-s001.pdf]

Supporting Information

Table of Contents

S1. ^{1}H NMR spectrum of ilimaquinone in $CDCl_3$ at 400 MHz.

S2. ^{13}C NMR spectrum of ilimaquinone in $CDCl_3$ at 100 MHz.

S3. HSQC spectrum of ilimaquinone in $CDCl_3$.

S4. HMBC spectrum of ilimaquinone in $CDCl_3$.

S5. ^{1}H–^{1}H COSY spectrum of ilimaquinone in $CDCl_3$.

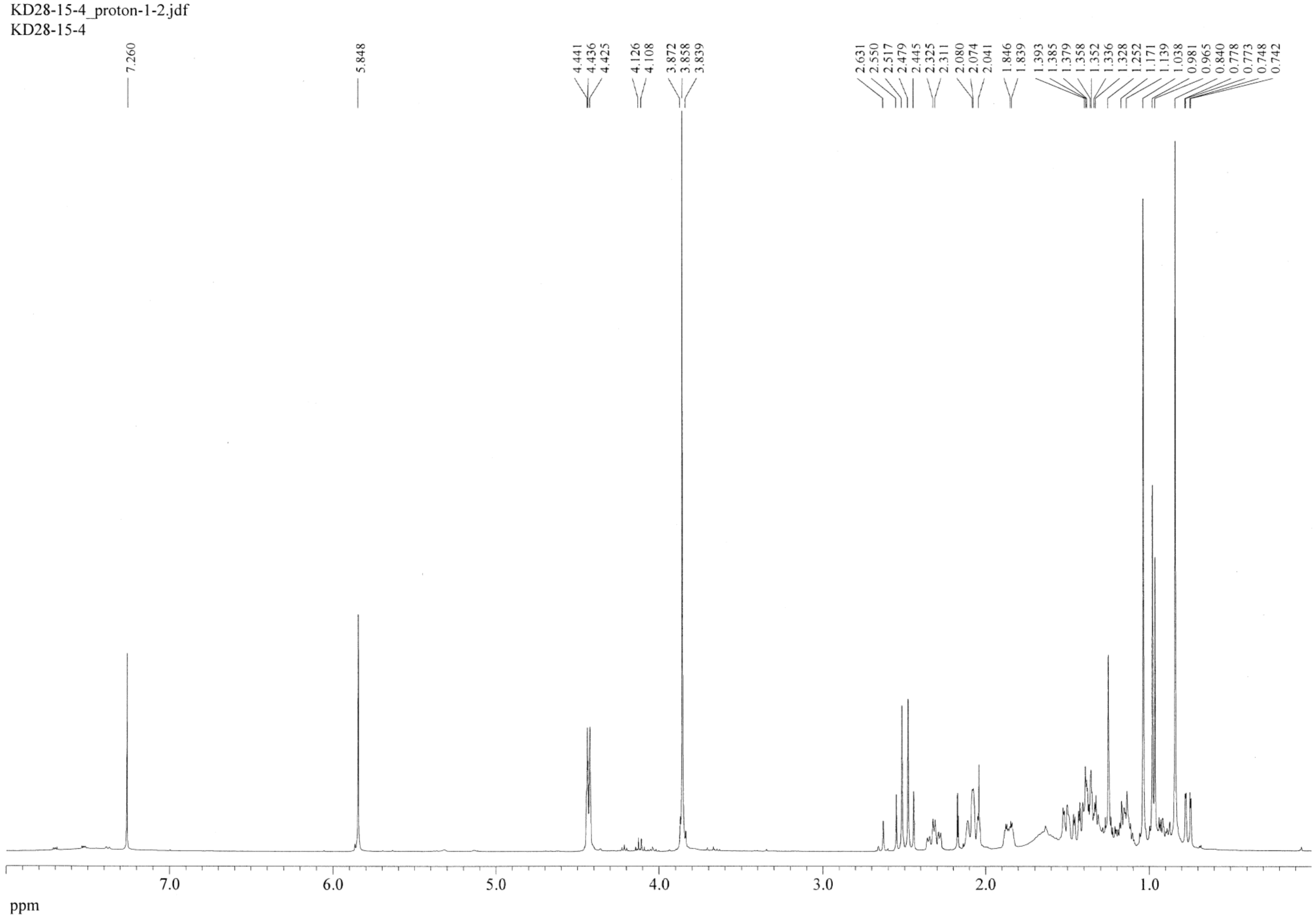

S1. ^{1}H NMR spectrum of ilimaquinone in $CDCl_3$ at 400 MHz.

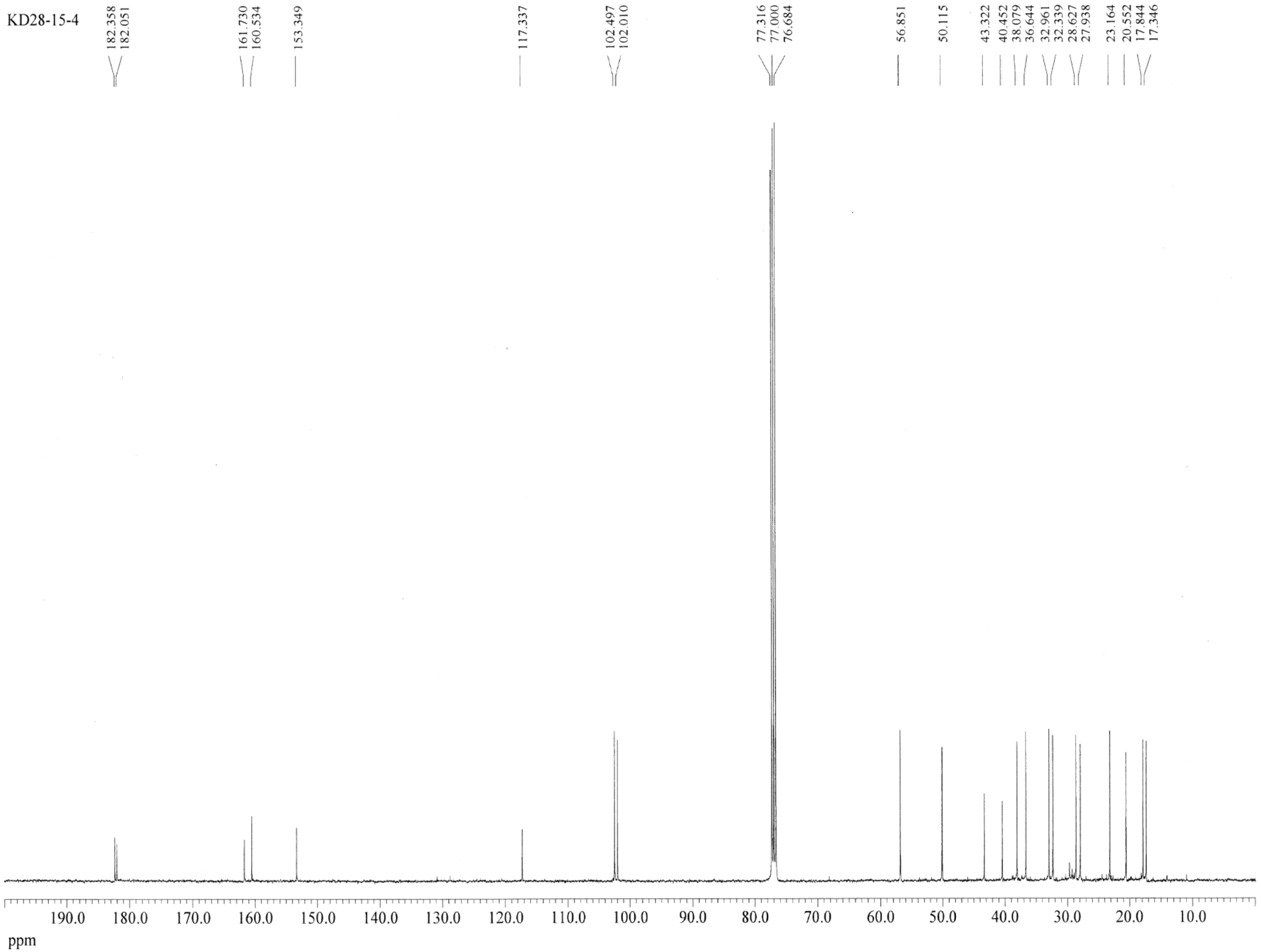

S2. ^{13}C NMR spectrum of ilimaquinone in $CDCl_3$ at 100 MHz.

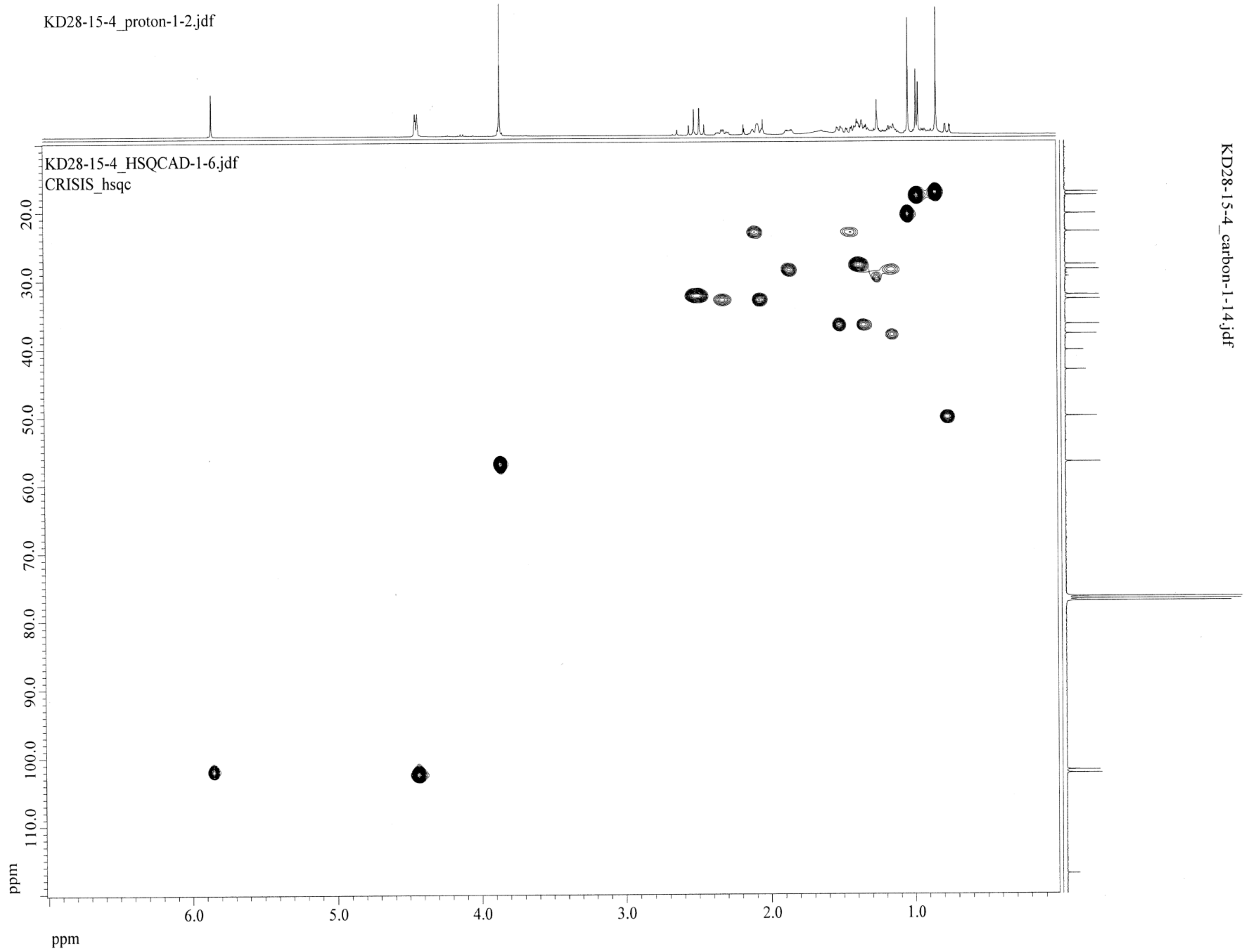

S3. HSQC spectrum of ilimaquinone in $CDCl_3$.

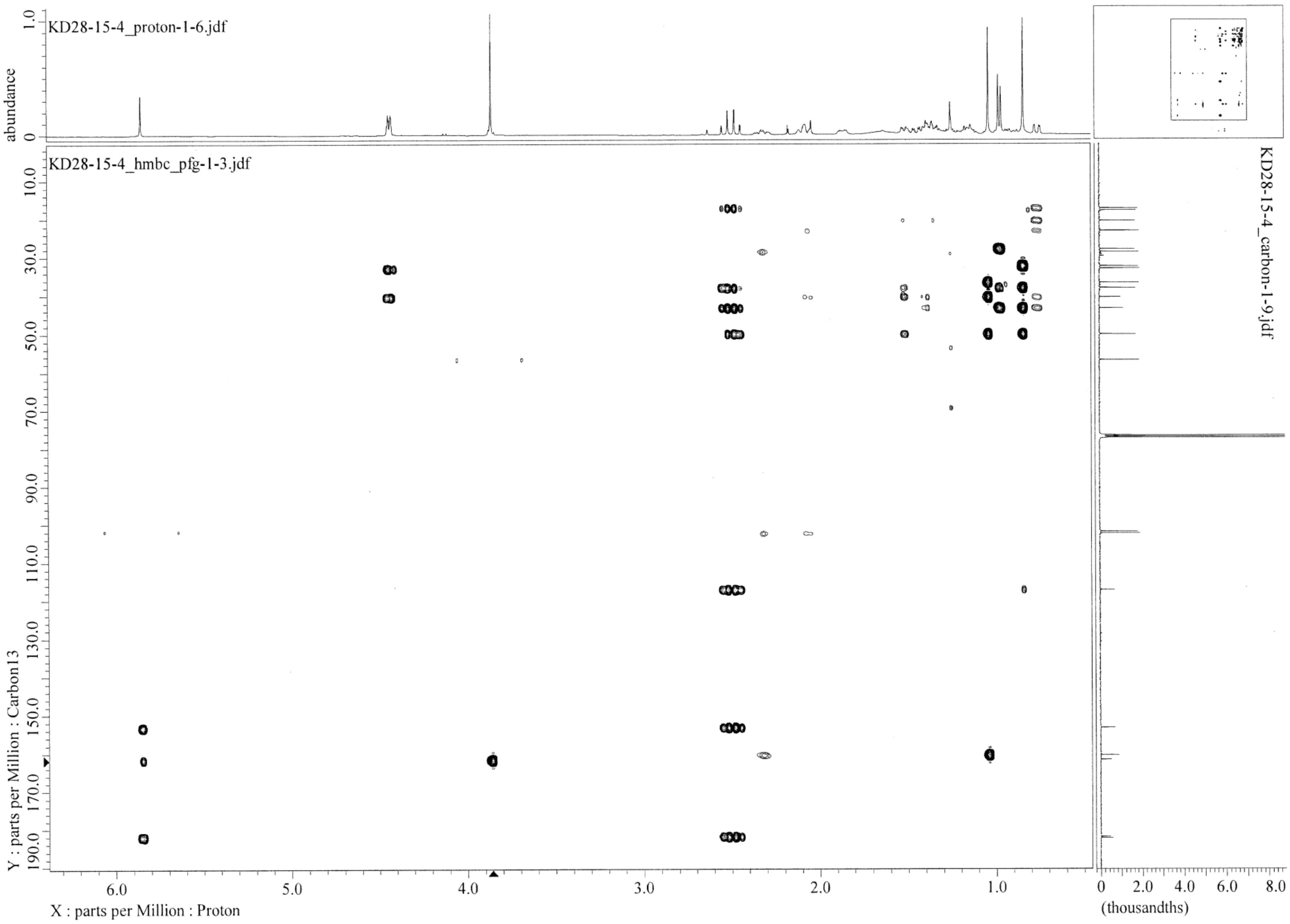

S4. HMBC spectrum of ilimaquinone in $CDCl_3$.

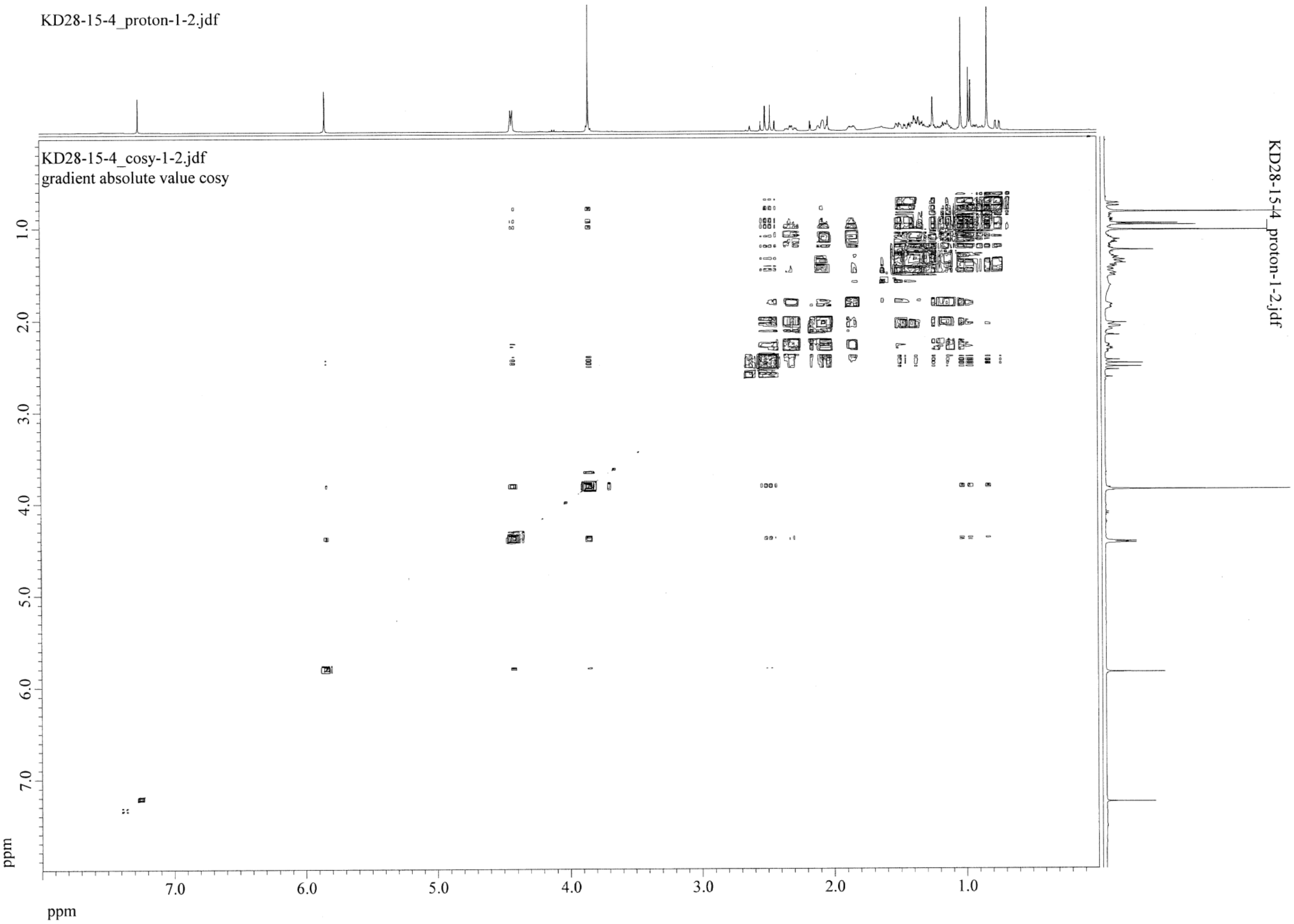

S5. ^{1}H–^{1}H COSY spectrum of ilimaquinone in $CDCl_3$.
